# Supplementary material for: One-pot H/D exchange and low-coordinated iron electrocatalyzed deuteration of nitriles in D2O to α,β-deuterio aryl ethylamines
Source: Nat Commun. 2022 Oct 10;13:5951. doi: 10.1038/s41467-022-33779-8 (PMC9550836; doi:10.1038/s41467-022-33779-8)
Supplement: Supplementary file 1 — Supplementary Information [file 41467_2022_33779_MOESM1_ESM.docx]

**­** Supplementary Information

**One-pot H/D exchange and low-coordinated iron electrocatalyzed deuteration of nitriles in D_2_O to *α*,*β*-deuterio aryl ethylamines**

Li et al.

**Contents**

**Supplementary Figure 1.** Representative examples of drug molecules.

**Supplementary Figure 2.** Deuterated experiment.

**Supplementary Figure 3.** Reaction setup.

**Supplementary Figure 4.** Standard calibration curves for quantitative analysis of the model reaction with dodecane as an internal standard.

**Supplementary Figure 5.** Calculated and experimental Pourbaix diagrams of Fe.

**Supplementary Figure 6.** O 1*s* XPS spectra of *α*-Fe_2_O_3_.

**Supplementary Figure 7.** In situ XRD study on the formation of Fe from *α*-Fe_2_O_3_.

**Supplementary Figure 8.** Experimental and simulated XAFS spectra of LC-Fe at the Fe K-edge.

**Supplementary Figure 9.** Linear sweep voltammetry (LSV) studies of LC-Fe under different conditions.

**Supplementary Figure 10.** Comparisons of different adsorption configurations of **1f** and **2f_Int_** on the LC-Fe(110) surface.

**Supplementary Figure 11.** N 1*s* XPS spectra of LC-Fe before and after treatments with **1a** and **2a**.

**Supplementary Figure 12.** The calculated Δ*G*_H*_ over Fe foil and LC-Fe.

**Supplementary Figure 13.** Possible reaction mechanism for electroreductive hydrogenation of nitriles with H_2_O over the LC-Fe cathode.

**Supplementary Figure 14.** The trapping of imine intermediate during electrocatalytic hydrogenation of benzonitrile with H_2_O.

**Supplementary Figure 15.** HR-MS analysis of the spin-trapping experiment of 1a electroreduction with H_2_O by using DMPO as a trapping agent.

**Supplementary Figure 16.** Qualitative analysis of hydrogenated products of electrocatalytic hydrogenation of 2-(4-ethynylphenyl)acetonitrile over LC-Fe by GC-MS.

**Supplementary Figure 17.** Examples of drug molecules.

**Supplementary Figure 18.** The correct calibration curves with internal standard dodecane.

**Supplementary Table 1.** Reaction optimization.

**Supplementary Table 2.** EXAFS fitting parameters at the Fe K-edge.

**Supplementary Table 3.** Substrate scope of electrocatalytic hydrogenation of aryl acetonitriles and benzonitriles over the LC-Fe cathode.

**Supplementary Notes 1-11.**

**NMR spectra of 2a to 2aab, 2-(4-methoxyphenyl)acetamide, and 2-(4-methoxyphenyl)acetic acid.**

**Supplementary References (1-15).**

**Supplementary Figures**

**Supplementary Figure 1.** **Representative examples of drug molecules.** Biologically active compounds containing the aryl ethylamine backbones.

**Supplementary Figure 2.** **Deuterated experiment.** *α*-C−H to *α*-C−D exchange of 0.1 mmol of **1a** with the assistance of KOH or K_2_CO_3_.


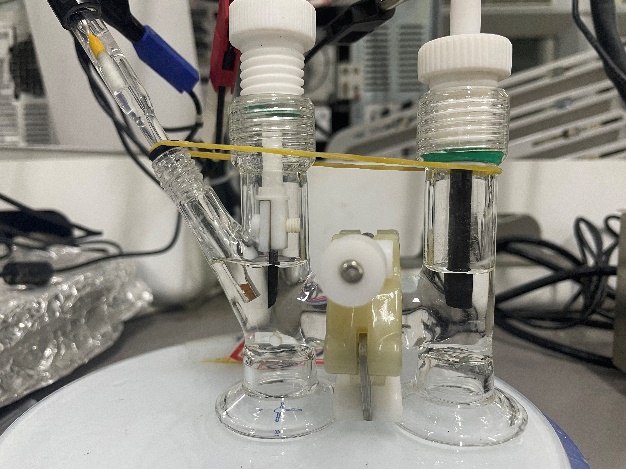


**Supplementary Figure 3.** **Reaction setup.** Reaction setup for electrocatalytic hydrogenation and deuteration of nitriles over the LC-Fe cathode.

**Supplementary Figure 4.** **Standard calibration curves for quantitative analysis of the model reaction with dodecane as an internal standard.** **a** *p*-methoxyphenylacetonitrile (**1a**), **b** *p*-methoxyphenethylamine (**2a**), and **c** *p*-methoxyphenylacetamide.

**Supplementary Figure 5.** **Calculated and experimental Pourbaix diagrams of Fe.** **a** The calculated and **b** experimental Pourbaix diagrams of Fe constructed with aqueous ion concentrations 10^-6^ M at 25 °C. Reproduced with permission Ref. 1 (Wang, Z. et al. Predicting aqueous stability of solid with computed Pourbaix diagram using SCAN functional. npj Comput. Mater. 2020, 6, 160), Copyright 2020, Springer Nature.

**Supplementary Figure 6.** **O 1*s* XPS spectra of *α*-Fe_2_O_3_.** In the O 1*s* XPS spectra, three peaks at 530.17 (predominant peak), 531.71, and around 535.35 eV (broad peak) originate from the lattice oxygen, the hydroxyl groups (OH^−^), and the adsorbed water molecules (H_2_O_ads_).^2^

**Supplementary Figure 7.** **In situ XRD study on the formation of Fe from *α*-Fe_2_O_3_.** The characteristic peaks of *α*-Fe_2_O_3_ disappear and the peaks belonging to the Fe(OH)_2_ become prominent after a period of electrolysis. With the electrolysis going on, the peaks of Fe(OH)_2_ vanish, and the characteristic peaks corresponding to cubic Fe raise, which is the final and stable phase of the reduced sample.

**Supplementary Figure 8.** **Experimental and simulated XAFS spectra of LC-Fe at the Fe K-edge.** The *χ*(k) data weighted by k^3^.

**Supplementary Figure 9.** **Linear sweep voltammetry (LSV) studies of LC-Fe under different conditions.** Reaction conditions: 8 mL anhydrous *N*,*N*-dimethylformamide (DMF), 0.2 M tetrabutylammonium tetrafluoroborate (TBAPF_4_), at a scan rate of 5 mV s^-1^, LC-Fe cathode, RT, with and without **1a** (0.1 mmol) and H_2_O (100 μL).

**Supplementary Figure 10.** **Comparisons of different adsorption configurations of 1f and 2f_Int_ on the LC-Fe(110) surface. a** Different adsorption configurations of **1f**. **b** Different adsorption configurations of **2f_Int_**. From the comparisons, **1f** and **2f_Int_** have prioritized adsorptions on LC-Fe via co-adsorption of aryl ring, C≡N, and C=N groups.

**Supplementary Figure 11.** **N 1*s* XPS spectra of LC-Fe before and after treatments with 1a and 2a.** A stronger N 1*s* signal in the N 1*s* XPS spectrum when treating LC-Fe with **1a** under our reaction conditions for 1 h. However, nearly no adsorption of **2a** on LC-Fe is observed. This may suggest the stronger adsorption of nitrile substrates on the LC-Fe surface than that of amine products.

**Supplementary Figure 12.** **The calculated Δ*G*_H*_ over Fe foil and LC-Fe.** The Gibbs free energy (Δ*G*_H*_) for H* formation on LC-Fe is more negative than that on Fe foil.

**Supplementary Figure 13.** **Possible reaction mechanism** **for electroreductive hydrogenation of nitriles with H_2_O over the LC-Fe cathode.** The main reaction course is shown involving the adsorption and desorption of reaction species on the LC-Fe surface.

**Supplementary Figure 14.** **The trapping of imine intermediate during electrocatalytic hydrogenation of benzonitrile with H_2_O.** Reaction conditions: benzonitrile (0.15 mmol), phenylmethanamine (0.15 mmol), LC-Fe NPs/CP cathode (working area: 1.0 cm^2^), 1.0 M KOH/Diox (v/v, 7:1, 8 mL), RT. The qualitative analytical results by GC-MS were reported.

**Supplementary Figure 15.** **HR-MS analysis of the spin-trapping experiment of 1a electroreduction with H_2_O by using DMPO as a trapping agent.** As illustrate in Supplementary Fig. 15, two peaks at *m/z* 116.0590 and 117.1018 belong to the HR-MS of DMPO-H (the theoretically calculated HR-MS for [M_(DMPO-H)+H_· + H]^+^ (C_6_H_14_NO^+^): *m/z* 116.1075 (100.0%), 117.1109 (6.5%)). Reaction conditions: **1a** (0.15 mmol), in a mixed solution of 1.0 M KOH in H_2_O/dioxane (3:1 v/v, 8 mL), LC-Fe cathode (working area: 1.0 cm^2^), at −1.2 V, 1 h.

**Supplementary Figure 16.** **Qualitative analysis of hydrogenated products of electrocatalytic hydrogenation of 2-(4-ethynylphenyl)acetonitrile over LC-Fe by GC-MS.** Reaction conditions: 2-(4-ethynylphenyl)acetonitrile (0.1 mmol), LC-Fe NPs/CP cathode (working area: 1.0 cm^2^), 1.0 M KOH/Diox (v/v, 3:1, 8 mL), RT. The qualitative analytical results by GC-MS were reported.

**Supplementary Figure 17.** **Examples of drug molecules.** The representative drugs containing the aryl ethylamine and benzylamine backbones which are in Fig 5.

**Supplementary Figure 18.** **The correct calibration curves with internal standard dodecane.** The correct calibration curves for quantitative analysis of nitriles which are in Fig. 5.

**Supplementary Table 1** Reaction optimization*^a^*

| entry | cathode | potential / V vs Hg/HgO | conversation / % of **1a***^b^* | selectivity / % of **2a***^b^* |
| --- | --- | --- | --- | --- |
| **1** | **LC-Fe NPs/CP** | **-1.2** | **97** | **97** |
| 2 | Fe foil | -1.4 | 76 | 64 |
| 3 | Pt foil | -1.4 | 81 | 40 |
| 4 | Pd foil | -1.4 | 80 | 25 |
| 5 | Mo foil | -1.4 | 47 | 12 |
| 6 | Cu foil | -1.4 | 40 | 10 |
| 7 | Ni foil | -1.4 | 43 | 7 |
| 8 | carbon paper (CP) | -1.4 | 29 | n.d.*^c^* |
| 9 | LC-Fe NPs/CP | -1.0 | 39 | n.d.*^c^* |
| 10 | LC-Fe NPs/CP | -1.1 | 73 | 77 |
| 11 | LC-Fe NPs/CP | -1.15 | 91 | 96 |
| 12 | LC-Fe NPs/CP | -1.25 | 97 | 97 |
| 13 | LC-Fe NPs/CP | -1.3 | 97 | 97 |

*^a^*Reaction conditions: **1a** (0.1 mmol), a mixed solvent of 1.0 M KOH/dioxane (3:1 v/v, 8 mL), RT, -1.2 V vs Ag/AgCl, 8 h. *^b^*Determined with GC using dodecane as the internal standard. *^c^*n.d. = Not detected.

**Supplementary Table 2** EXAFS fitting parameters at the Fe K-edge（Ѕ_0_^2^ = 0.71）

| Sample | Path | C.N. | *R* (Å) | *σ*^2^×10^3^ (Å^2^) | Δ*E* (eV) | *R* factor |
| --- | --- | --- | --- | --- | --- | --- |
| Fe foil | Fe-Fe | 8* | 2.47±0.01 | 4.6±0.8 | 7.2±1.2 | 0.002 |
|  | Fe-Fe | 6* | 2.85±0.01 | 5.9±1.7 | 5.8±2.6 |  |
| experiment | Fe-Fe | 6.1±1.9 | 2.48±0.02 | 2.2±2.1 | 11.2±5.1 | 0.018 |
|  | Fe-Fe | 4.1±4.0 | 2.88±0.03 | 2.9±6.2 |  |  |

C.N.: coordination number; *R*: bond distance; *σ*^2^: Debye-Waller factors; Δ*E*: the inner potential correction; *R* factor: goodness of fit. * Fitting with fixed parameter. The *k*^3^ weighting, k-range of 2 ~ 12 Å^-1^ and R range of 1.6 ~ 3.6 Å were used for the fitting.

**Supplementary Table 3** Substrate scope of electrocatalytic hydrogenation of aryl acetonitriles and benzonitriles over the LC-Fe cathode.

Reaction conditions: substrate (0.1 mmol), LC-Fe cathode (working area: 1.0 cm^2^), a mixed solution of 1.0 M KOH/dioxane (3:1 v/v, 8 mL), RT, 8 h. Conversion of substrates and isolated yields of the hydrochloride of primary amines (in parentheses) were reported.

**Supplementary Note 1**

**Reagents and chemicals.** All chemicals used in experiments are analytical pure and without further purification.

**Pretreatment of carbon fiber paper (CP).** CP (10 cm × 10 cm) was cut into several pieces with an average area of 3.0 cm × 1.0 cm of each piece. Then, they were ultrasonicated with acetone for 10 minutes to remove organics on the surface and washed with deionized (DI) water several times to remove acetone. After that, CP was immersed into the mixed solvents (1:1:1, v/v/v) containing DI water, H_2_SO_4_ (98% wt%), and HNO_3_ (68% wt%). The mixture was heated to 60 ºC in an oil bath and maintained for 24 h to improve its hydrophilicity.

**Pretreatment of Fe foil, Cu foil, Mo foil, and Pt foil.** All the metallic materials used in this paper were treated in the same way. After cutting into pieces of 3.0 cm × 1.0 cm, the materials were sonicated with acetone, 3.0 M of HCl aqueous solution, and DI water several times, respectively, to remove impurities.

**Synthesis of Pd on CP.** CP-supported Pd cathode was synthesized according to a modified method via electrodeposition from Pd^2+^ containing electrolyte.^3^ The electrodeposition of Pd was conducted in a general three-electrodes cell system using a piece of carbon fiber paper as the working electrode (exposure area is 1.0 cm^2^), a carbon rod as the counter electrode, and an Ag/AgCl (3.0 M KCl) as the reference electrode. The electrochemical deposition of Pd is performed at 0.0 V vs. Ag/AgCl for 600 s in a 40 mM PdCl_2_ aqueous solution.

**Supplementary Note 2**

**GC measurements.** In this work, conversion (Conv.) was referred to the substrate, and selectivity (Sele.) was referred to the primary amine product. Gas chromatography (GC) was used for quantification analysis of the hydrogenation reactions according to the standard calibration curves (one example is seen in Supplementary Fig. 4). When the reaction was finished, the products at the cathode were extracted with dichloromethane (DCM) four times and dried with anhydrous Na_2_SO_4_. Then, the DCM was removed under reduced pressure on the rotary evaporator. The residuals were re-dissolved with ethyl acetate (EA) and then analyzed by GC. The spectra were obtained with a gas chromatograph (Agilent 7890) equipped with the HP-5 capillary column (30 m × 250 μm), thermal conductivity (TCD), and a flame ionization detector (FID). The injection temperature was set at 300 °C. Nitrogen was used as the carrier gas at 1.5 mL min^-1^. The initial column temperature was 50 °C and increases to 200 °C at 10 °C min^-1^ at the first step, then increases to 220 °C at 5 °C min^-1^ and finally to 250 °C at 10 °C min^-1^, keeping 250 °C for 5 min. A 1.0 μL of the sample was taken out for detection by GC.

**Supplementary Note 3**

**Equations for calculating the Conv., Sele., Yield, and deuterated ratios.** To be more specific, “Conv.” was calculated by dividing the amount (millimoles) of the consumed substrate by originally added millimoles, and “ Sele.” was calculated by dividing the obtained millimoles of the product by the consumed millimoles of the substrate. In addition, isolated yields of the products in Fig. 5 and Supplementary Table 2 were provided, which were calculated by dividing the obtained millimoles of the hydrogenated or deuterated products by the initially added substrates. The equations were found as follows (1) – (3):

$$\text{Conv. }\text{(\%)}\text{ = }\frac{\text{n}\text{ }\left( \text{consumed substrates} \right)}{\text{n}\text{ }\left( \text{initial substrates} \right)}\text{ × 100\% (1)}$$

$$\text{Sele}\text{.}\text{ (\%) = }\frac{\text{n}\text{ (}\text{obtained products}\text{)}}{\text{n}\text{ (consumed substrates)}}\text{×}\text{ }\text{100\%}\text{ }\text{(2)}$$

$$\text{Y}\text{ield (\%)}\text{ =}\frac{\text{n}\text{ }\text{(}\text{obtained products}\text{)}}{\text{n}\text{ (}\text{initally added substrates }\text{)}}\text{ }\text{×}\text{ }\text{100\%}\text{ }\text{(3)}$$

In addition, deuterated ratios of the deuterated products were determined by ^1^H NMR according to the equations below (4) – (5).

$$\text{ }\text{Deuterium incorporation at }\text{α}\text{-position (\%) }$$

$$\text{ }\text{= 100\% }\text{–}\frac{\text{area (R-CH}\text{2}\text{-C}\text{D}\text{2}\text{-NH}\text{2}\text{·HCl)}}{\text{2}}\text{ }\text{×}\text{ }\text{100\% }\text{ }\text{(4)}$$

$$\text{ }\text{Deuterium incorporation at }\text{β}\text{-position (\%) }$$

$$\text{ }\text{= 100\% }\text{–}\frac{\text{area (R-C}\text{D}\text{2}\text{-CH}\text{2}\text{-NH}\text{2}\text{·HCl)}}{\text{2}}\text{ ×}\text{ }\text{100\% }\text{ }\text{(}\text{5}\text{)}$$

**Supplementary Note 4**

**Characterizations of *α*-Fe_2_O_3_.** The scanning electron microscopy (SEM) image (Fig. 2b) reveals that Fe_2_O_3_ nanorods grow uniformly on carbon fiber paper (CP). All the diffraction peaks in the X-ray diffraction (XRD) pattern are indexed to *α*-Fe_2_O_3_ (JCPDS NO. 36-0664, Fig. 2e). X-ray photoelectron spectroscopy (XPS) is further adapted to confirm the valence state of Fe and O. In the Fe 2*p* XPS spectra (Fig. 2f), two characteristic peaks at 711.2 eV and 724.8 eV are assigned to Fe^3+^ 2*p*_1/2_ and Fe^3+^ 2*p*_3/2_, respectively, with a spin energy separation of 13.6 eV. Two peaks located at around 732.8 and 719.5 eV are the satellite peaks of Fe 2*p*^2^.

**Supplementary Note 5**

**Computational details.** All the computations were performed based on the density functional theory (DFT) methods, as implemented in the plane wave set Vienna ab initio Simulation Package (VASP) code.^4,5^ The exchange-correlation functional in the Perdew-Burke-Ernzerhof (PBE) form within a generalized gradient approximation (GGA) was used.^6^ Spin polarization was considered in all calculations. To better describe the on-site coulomb (*U*) correlation of the localized 3*d* electrons for transition metal Fe and Cu, the DFT + *U* method with *U* - *J* = 3.29 eV for Ti and *U* - *J* = 3.87 eV for Cu was adopted.^7,8^ To better describe the weak long-distance van der Waals (vdWs) interaction, an empirical dispersion corrected DFT method (DFT-D_3_) was carried out.^9^ A kinetic-energy cut-off of 500 eV was set. The convergence threshold for the iteration in the self-consistent field (SCF) was set as 10^-4^ eV. The geometry optimization within the conjugate gradient method was performed with forces on each atom less than 0.05 eV Å^-1^. Fe (110) surface was modeled with three atomic layers. To prevent periodic image interactions, a large vacuum layer of 15 Å was inserted in the *z*-direction. The bottom atomic layer was fixed while other layers and the adsorbates were fully relaxed during structural optimizations. The Brillouin zone was sampled by a *k*-point mesh of 4 × 4 × 1.

The adsorption energy (*E*_ads_) of adsorbates was calculated as follows:

*E*_ads_ *= E*_adsorbate/slab_ *– E*_slab_ *– E*_adsorbate_ (6)

where *E*_adsorbate/slab_ was the total energy of adsorbate on the surface; *E*_slab_ and *E*_adsorbate_ were the total energies of the surface and the free adsorbate, respectively.

The reaction free energy change can be obtained with the following equation:

Δ*G* = Δ*E* + Δ*E*_ZPE_ – TΔ*S*  (7)

where Δ*E* was the total energy difference between the products and the reactants of each reaction step, and Δ*E*_ZPE_ and Δ*S* were the differences in zero-point energy and entropy, respectively. The zero-point energy of free molecules and adsorbates were obtained from the vibrational frequency calculations. The free energy change of each step that involved an electrochemical proton-electron transfer was described by the computational hydrogen electrode (CHE) model proposed by Nørskov et al.^10^ In this technique, zero voltage was defined based on the reversible hydrogen electrode, in which the reaction was defined to be in equilibrium at zero voltage, at all values of pH, at all temperatures, and with H_2_ at 101,325 Pa pressure. Therefore, in the CHE model, the free energy of a proton-electron pair was equal to half of the free energy of gaseous hydrogen at a potential of 0 V.

**Supplementary Note 6**

**EPR experiments of electrocatalytic hydrogenation of 1a.** The hydrogen radicals are trapped by the addition of 5,5-dimethyl-1-pyrroline-*N*-oxide (DMPO) in a mixed solution of 1.0 M KOH/dioxane (3:1 v/v, 8 mL). A LC-Fe working electrode and a Hg/HgO reference electrode were put into the cathode chamber, and a graphite rod counter electrode was inserted into the anode chamber. Chronoamperometry was carried out at a given constant potential of −1.2 V vs. Hg/HgO for 5 mins with or without 0.1 mmol **1a** in a mixed solution of 1.0 M KOH/dioxane (3:1 v/v, 8 mL). Then, 0.1 mmol 5,5-dimethyl-1-pyrroline-*N*-oxide (DMPO) dissolved in dioxane was added and stirred for 1 min. After that, the solution was quickly taken out for the EPR test.

**Supplementary Note 7**

**HR-MS analysis of the spin-trapping experiment of 1a electroreduction with H_2_O by using DMPO as a trapping agent.** Supplementary Fig. 15 displays that two peaks at *m/z* 116.0590 and 117.1018 belong to the HR-MS of DMPO-H (the theoretically calculated HR-MS for [M_(DMPO-H)+H_· + H]^+^ (C_6_H_14_NO^+^): *m/z* 116.1075 (100.0%), 117.1109 (6.5%)). Whereas, the HR-MS of the adduct of DMPO with carbon radical **I** (denote as DMPO-**I**) is found at *m/z* 263.1744 and 264.1781 (the theoretically calculated HR-MS for [M_(DMPO-_**_I_**_)+H_· + H]^+^ (C_15_H_23_N_2_O_2_^+^): *m/z* 263.1755 (100.0%), 264.1788 (16.2%), Fig. 4e). And, the HR-MS of the adduct of DMPO with carbon radical **III** (denote as DMPO-**III**) is found at *m/z* 265.1902 and 266.1752 (the theoretically calculated HR-MS for [M_(DMPO-_**_III_**_)+H_· + H]^+^ (C_15_H_25_N_2_O_2_^+^): *m/z* 265.1911 (100.0%), 266.1945 (16.2%), Fig. 4e). These results provide solid evidence to identify the involved radical species during electrocatalytic hydrogenation of **1a**, further rationalizing our proposed mechanism.

**Supplementary Note 8**

**General procedure to get the amines hydrochloride.** When the electroreduction of nitriles finished, the reaction mixture was extracted three times with**DCM. The combined DCM was dried over anhydrous Na_2_SO_4_** and then was removed under reduced pressure to obtain the crude products containing the deuterated products. After that, the crude products were re-dissolved in 3 mL DCM solvent and then was treated with a 3.0 M solution of HCl in cyclopentylmethyl ether to precipitate the solid products, which were filtered for calculating the isolated yields.

**Supplementary Note 9**

The NMR spectra and data of the hydrochloride of amine products were provided. When using *d*_6_-DMSO as the solvent for NMR tests, the peaks at 2.5 ppm and 3.3 ppm in ^1^H NMR spectra were assigned to the hydrogen signal of *d*_6_-DMSO and H_2_O, respectively. And, the broad peak centered at around 2.5 ppm in ^2^H NMR spectra belonged to the deuterium signal of *d*_6_-DMSO. Seven peaks centered at 39.52 ppm in ^13^C NMR spectra were assigned to the carbon signals of *d*_6_-DMSO. When using CDCl_3_ as the solvent for NMR tests, the peak at 7.26 ppm in ^1^H NMR spectra was assigned to the hydrogen signal of CDCl_3_. And, the broad peak at 7.75 ppm in ^2^H NMR spectra belonged to the deuterium signal of CDCl_3_. Three peaks centered at 77 ppm in ^13^C NMR spectra were assigned to the carbon signals of CDCl_3_. And, the broad peak at 1.94 ppm in ^2^H NMR spectra belonged to the deuterium signal of CD_3_CN. Three peaks at 1.79 and 118.89 ppm in ^13^C NMR spectra were assigned to the carbon signals of CD_3_CN. Additionally, some carbon atoms are missed in the ^13^C spectra of the deuterated products due to the presence of D, which are consistent with the reported literature.^11-13^ Furthermore, the GC-MS data of amine products were provided. The *m*/*z* differences between the theoretically calculated data and the tested data by GC-MS of the deuterated products were ascribed to high activity of the N−D bond causing H/D exchange during the tests.

**Supplementary Note 10**

**The synthetic procedure of *d*_4_-*Melatonin*.** After the electroreductive deuteration of **1aab** was finished, the reaction mixture was extracted three times with**DCM. The combined DCM was dried over anhydrous Na_2_SO_4_** and then was removed under reduced pressure to obtain the crude products containing the deuterated product **2aab**. Next, the crude products were dissolved in 3 mL anhydrous DCM with adding triethylamine (0.23 mmol, 1.5 equivalent) to form a homogeneous solution. Then carboxy chloride (0.20 mmol, 1.3 equivalent) was slowly added to the solution at 0 °C. Then, the reaction mixture was stirred at room temperature for 30 min. After that, the reaction was quenched with saturated ammonium chloride solution and extracted with EA three times. The combined EA organic phase was washed with brine, dried with **anhydrous** Na_2_SO_4_, and then concentrated in vacuo to obtain the crude *d_4_-Melatonin*. The crude *d_4_-Melatonin* was separated by the thin-layer chromatography (TLC) plate to give the pure product.^14^

**Supplementary Note 11**

**The synthetic procedure of *d*_4_-*Komavine*.** After the electroreductive deuteration of **1aaa** was finished, the reaction mixture was extracted three times with**DCM. The combined DCM was dried over anhydrous Na_2_SO_4_** and then was removed under reduced pressure to obtain the crude products containing the deuterated product **2aaa**. Next, Pd(OH)_2_/C (20 wt%, 0.015 mmol, 0.1 equivalent) and sodium formate (0.23 mmol, 1.5 equivalent) were added into a round bottom (20 mL) charged with a magnetic stir bar, which was evacuated and back-filled with argon. Subsequently, the crude products containing **2aaa** were dissolved into toluene (0.2 mL), which were added to the round bottom with additional 1 mL DI water. The reaction mixture was stirred in a preheated oil bath at 130 ^o^C for 12 h, and then cooled to room temperature. The mixture was extracted with DCM (3 × 20 mL) and the combined organic extracts were washed with brine, dried over **anhydrous** Na_2_SO_4_, concentrated, and purified by the TLC plate to give the pure product.^15^

**NMR, GC-MS, and HR-MS data**

**^1^H NMR** (400 MHz, DMSO-*d*_6_) *δ* [ppm] 8.07 (s, 2.59H), 7.17 (d, *J* = 8.0 Hz, 2H), 6.89 (d, *J* = 4.0 Hz 2H), 3.72 (s, 3H), 2.96 (t, *J* = 8.0 Hz, 2H), 2.84 – 2.79 (t, *J* = 8.0 Hz, 2H); **^13^C NMR** (101 MHz, DMSO-*d*_6_) *δ* [ppm] 158.09, 129.70 (2C), 129.27, 114.03 (2C), 55.07, 32.09 (2C); **GC–MS** (EI) [M]^-^ 151.08, theoretical value for C_9_H_13_NO *m/z* 151.21.

**^1^H NMR** (400 MHz, DMSO-*d*_6_) *δ* [ppm] 8.24 (s, 3H), 7.31 (t, *J*_1_ = 8.0 Hz, 2H), 7.14 (t, *J* = 8.9 Hz, 2H), 2.99 (t, *J* = 8.0 Hz, 2H), 2.90 (t, *J* = 8.0 Hz, 2H); **^13^C NMR** (101 MHz, DMSO-*d*_6_) *δ* [ppm] 161.17 (d, *J* = 243.4 Hz), 133.66, 130.61 (d, *J* = 8.1 Hz, 2C), 115.33 (d, *J* = 21.2 Hz, 2C), 32.06 (2C); **GC–MS** (EI) [M]^-^ 139.05, theoretical value for C_8_H_10_FN *m/z* 139.17.

**^1^H NMR** (400 MHz, DMSO-*d*_6_) *δ* [ppm] 8.23 (s, 3H), 7.38 (d, *J* = 8.0 Hz, 2H), 7.30 (d, *J* = 8.0 Hz, 2H), 2.99 (s, 2H), 2.90 (t, *J* = 8.0 Hz, 2H); **^13^C NMR** (101 MHz, DMSO-*d*_6_) *δ*[ppm] 136.52, 131.38, 130.67 (2C), 128.54 (2C), 32.18 (2C); **GC–MS** (EI) [M]^-^ 155.03, theoretical value for C_8_H_10_ClN *m/z* 155.63.

**^1^H NMR** (400 MHz, DMSO-*d*_6_) *δ* [ppm] 8.20 (s, 3H), 7.51 (d, *J* = 8.0 Hz, 2H), 7.24 (d, *J* = 4.0 Hz, 2H), 3.00 (t, *J* = 4.0 Hz, 2H), 2.88 (t, *J* = 4.0 Hz, 2H); **^13^C NMR** (101 MHz, DMSO-*d*_6_) *δ* [ppm] 136.90, 131.47 (2C), 131.07 (2C), 119.90, 32.27 (2C); **GC–MS** (EI) [M]^-^ 199.98, theoretical value for C_8_H_10_BrN *m/z* 200.08.

**^1^H NMR** (400 MHz, DMSO-*d*_6_) *δ* [ppm] 8.26 (s, 2.37H), 7.13 (s, 4H), 2.96 (s, 2H), 2.86 (d, *J* = 4.0 Hz, 2H), 2.26 (s, 3H); **^13^C NMR** (101 MHz, DMSO-*d*_6_) *δ* [ppm] 135.71, 134.38, 129.18 (2C), 128.52 (2C), 32.51 (2C), 20.66; **GC–MS** (EI) [M]^-^ 135.08, theoretical value for C_9_H_13_N *m/z* 135.21.

**^1^H NMR** (400 MHz, DMSO-*d*_6_) *δ* [ppm] 8.20 (s, 2.85H), 7.39 – 7.17 (m, 5H), 3.00 (t, *J* = 8.0 Hz, 2H), 2.90 (t, *J* = 8 Hz, 2H); **^13^C NMR** (101 MHz, DMSO-*d*_6_) *δ* [ppm] 137.49, 128.67 (2C), 128.64 (2C), 126.73, 32.96 (2C); **GC–MS** (EI) [M]^-^ 121.07, theoretical value for C_8_H_11_N *m/z* 121.18.

**^1^H NMR** (400 MHz, DMSO-*d*_6_) *δ* [ppm] 11.06 (s, 0.94H), 8.23 (s, 2.91H), 7.57 (d, *J* = 8.0 Hz, 1H), 7.37 (d, *J* = 8.0 Hz, 1H), 7.24 (d, *J* = 4.0 Hz, 1H), 7.08 (dt, *J*_1_ = 8.0 Hz, *J*_2_ = 1.12 Hz, 1H), 6.99 (dt, *J*_1_ = 8.0 Hz, *J*_2_ = 0.99 Hz, 1H), 3.04 (d, *J* = 2.2 Hz, 4H); **^13^C NMR** (101 MHz, DMSO-*d*_6_) *δ* [ppm] 136.32, 126.85, 123.36, 121.16, 118.47, 118.16, 111.58, 109.57, 23.08 (2C); **GC–MS** (EI) [M]^-^ 160.09, theoretical value for C_10_H_12_N_2_ *m/z* 160.22.

**^1^H NMR** (400 MHz, DMSO-*d*_6_) *δ* [ppm] 8.08 (s, 2.28H), 6.93 – 6.83 (m, 2H), 6.75 (td, *J*_1_ = 4.0 Hz, *J*_2_ = 1.24 Hz, 1H), 3.74 (d, *J* = 4.0 Hz, 3H), 3.71 (d, *J* = 4.0 Hz, 3H), 2.99 (s, 2H), 2.81 (t, *J* = 4.0 Hz, 2H); **^13^C NMR** (101 MHz, DMSO-*d*_6_) *δ* [ppm] 148.82, 147.68, 129.71, 120.60, 112.53, 112.01, 55.56, 55.47, 32.58 (2C); **GC–MS** (EI) [M]^-^ 181.12, theoretical value for C_10_H_15_NO_2_ *m/z* 181.24.

**^1^H NMR** (400 MHz, DMSO-*d*_6_) *δ* [ppm] 8.52 (s, 2.87H), 7.43 (d, *J* = 4.0 Hz, 2H), 6.95 (d, *J* = 4.0 Hz, 2H), 3.92 (d, *J* = 4.0 Hz, 2H), 3.75 (s, 3H); **^13^C NMR** (101 MHz, DMSO-*d*_6_) *δ* [ppm] 159.37, 130.60 (2C), 126.03, 113.93 (2C), 55.23, 41.65; **GC–MS** (EI) [M]^-^ 137.06, theoretical value for C_8_H_11_NO *m/z* 137.18.

**^1^H NMR** (400 MHz, DMSO-*d*_6_) *δ* [ppm] 8.55 (s, 3H), 7.44 (t, *J* = 8.0 Hz, 2H), 7.28 (t, *J* = 4.0, 2H), 3.94 (s, 2H), 2.47 (s, 3H); **^13^C NMR** (101 MHz, DMSO-*d*_6_) *δ* [ppm] 138.68, 130.53, 129.72 (2C), 125.82 (2C), 41.71, 14.60; **GC–MS** (EI) [M]^-^ 153.04, theoretical value for C_8_H_11_NS *m/z* 153.24.

**^1^H NMR** (400 MHz, DMSO-*d*_6_) *δ* [ppm] 10.53 (s, 1.93H), 8.64 (s, 2.95H), 7.61 (d, *J* = 8.0 Hz, 2H), 7.42 (d, *J* = 8.0 Hz, 2H), 4.02 (q, *J* = 5.9 Hz, 2H); **^13^C NMR** (101 MHz, DMSO-*d*_6_) *δ* [ppm] 133.75, 132.32, 130.39 (2C), 123.31 (2C), 41.55; **GC–MS** (EI) [M]^-^ 122.06, theoretical value for C_7_H_10_N_2_ *m/z* 122.17.

**^1^H NMR** (400 MHz, DMSO-*d*_6_) *δ* [ppm] 8.72 (s, 2.93H), 8.14 – 7.77 (m, 4H), 7.61 (dd, *J* = 49.6, *J* = 6.9 Hz, 3H), 4.17 (d, *J* = 4.0 Hz, 2H); **^13^C NMR** (101 MHz, DMSO-*d*_6_) *δ* [ppm] 132.62 (2C), 131.75, 128.19, 127.99, 127.78, 127.70, 126.64, 126.61, 126.55, 42.30; **GC–MS** (EI) [M]^-^ 157.12, theoretical value for C_11_H_11_N *m/z* 157.22.

**^1^H NMR** (400 MHz, DMSO-*d*_6_) *δ* [ppm] 8.17 (s, 2.46H), 7.17 (d, *J* = 8.0 Hz, 2H), 6.88 (d, *J* = 8.0 Hz, 2H), 3.72 (s, 3H), 2.98 (s, 0.1H); **^13^C NMR** (101 MHz, DMSO-*d*_6_) *δ* [ppm] 158.11, 129.72 (2C), 129.17, 114.05 (2C), 55.08; **HR–MS** (ESI-TOF) found 156.1319, theoretical value for C_9_H_9_D_4_NO *m/z* [M+H]^+^ 156.1326.

**^2^H NMR** (400 MHz, DMSO-*d*_6_) *δ* [ppm] 2.89, 3.43, 8.01.

**^1^H NMR** (400 MHz, DMSO-*d*_6_) *δ* [ppm] 8.32 (t, *J* = 20.0 Hz, 1.89H), 7.12 (d, *J* = 4.0 Hz, 4H), 2.92 (s, 0.14H), 2.26 (s, 3H); **^13^C NMR** (101 MHz, DMSO-*d*_6_) *δ* [ppm] 135.71, 134.32, 129.19 (2C), 128.53 (2C), 20.69; **HR–MS** (ESI-TOF) found 140.1368, theoretical value for C_9_H_9_D_4_N *m/z* [M+H]^+^ 140.1377.

**^2^H NMR** (400 MHz, DMSO-*d*_6_) *δ* [ppm] 8.09, 3.41.

**^1^H NMR** (400 MHz, DMSO-*d*_6_) *δ* [ppm] 10.58 (s, 1.76H), 8.25 (t, *J* = 16 Hz, 2H), 7.38 – 7.35 (m, 4H), 2.97 (s, 0.12H); **^13^C NMR** (101 MHz, DMSO-*d*_6_) *δ* [ppm] 137.39, 130.40, 129.96 (2C), 123.54 (2C); **HR–MS** (ESI-TOF) found 141.1324, theoretical value for C_8_H_8_D_4_N_2_ *m/z* [M+H]^+^ 141.1330.

**^2^H NMR** (400 MHz, DMSO-*d*_6_) *δ* [ppm] 8.10, 2.95, 2.90.

**^1^H NMR** (400 MHz, DMSO-*d*_6_) *δ* [ppm] 9.38 (s, 0.38H), 8.04 (s, 1.29H), 7.03 (d, *J* = 8.0 Hz, 2H), 6.72 (d, *J* = 8.0 Hz, 2H); **^13^C NMR** (101 MHz, DMSO-*d*_6_) *δ* [ppm] 156.18, 129.62 (2C), 127.22, 115.38 (2C); **HR–MS** (ESI-TOF) found 142.1165, theoretical value for C_8_H_7_D_4_NO *m/z* [M+H]^+^ 142.1170.

**^2^H NMR** (400 MHz, DMSO-*d*_6_) *δ* [ppm] 7.93, 3.46.

**^1^H NMR** (400 MHz, DMSO-*d*_6_) *δ* [ppm] 8.30 (d, *J* = 20.5 Hz, 1.03H), 7.37 – 7.29 (m, 2H), 7.29 – 7.20 (m, 3H), 2.96 (s, 0.11H); **^13^C NMR** (101 MHz, DMSO-*d*_6_) *δ* [ppm] 137.42, 128.68 (2C), 128.66 (2C), 126.74; **HR–MS** (ESI-TOF) found 126.1213, theoretical value for C_8_H_7_D_4_N *m/z* [M+H]^+^ 126.1221.

**^2^H NMR** (400 MHz, DMSO-*d*_6_) *δ* [ppm] 8.10, 3.39, 2.95, 2.86.

**^1^H NMR** (400 MHz, DMSO-*d*_6_) *δ* [ppm] 8.01 (s, 0.56H), 7.32 – 7.28 (m, 2H), 7.17 – 7.13 (m, 2H), 2.94 (s, 0.11H); **^13^C NMR** (101 MHz, DMSO-*d*_6_) *δ* [ppm] 161.17 (d, *J* = 161.6 Hz), 133.68, 130.62 (d, *J* = 6.1 Hz, 2C), 115.33 (d, *J* = 14.1 Hz, 2C); **HR-MS** (ESI**–**TOF) found 144.1121, theoretical value for C_8_H_6_D_4_FN *m/z* [M+H]^+^ 144.1127.

**^2^H NMR** (400 MHz, DMSO-*d*_6_) *δ* [ppm] 8.06, 3.38, 2.94, 2.82.

**^1^H NMR** (400 MHz, DMSO-*d*_6_) *δ* [ppm] 8.08 (s, 2.97H), 7.38 (d, *J* = 4.0 Hz, 2H), 7.30 (d, *J* = 6.0 Hz, 2H), 2.97 (s, 0.16H); **^13^C NMR** (101 MHz, DMSO-*d*_6_) *δ* [ppm] 136.35, 131.43, 130.69 (2C), 128.56 (2C); **HR–MS** (ESI-TOF) found 160.0823, theoretical value for C_8_H_6_D_4_ClN *m/z* [M+H]^+^ 160.0831.

**^2^H NMR** (400 MHz, DMSO-*d*_6_) *δ* [ppm] 2.93, 2.83.

**^1^H NMR** (400 MHz, DMSO-*d*_6_) *δ* [ppm] 8.15 (s, 1.73H), 7.52 (d, *J* = 4.0 Hz, 2H), 7.25 (d, *J* = 2.0 Hz, 2H), 2.97 (s, 0.15H); **^13^C NMR** (101 MHz, DMSO-*d*_6_) *δ* [ppm] 137.42, 128.68 (2C), 128.66 (2C), 126.74; **HR–MS** (ESI-TOF) found 204.0319, theoretical value for C_8_H_6_D_4_BrN *m/z* [M+H]^+^ 204.0326.

**^2^H NMR** (400 MHz, DMSO-*d*_6_) *δ* [ppm] 7.98, 3.45, 3.39.

**^1^H NMR** (400 MHz, DMSO-*d*_6_) *δ* [ppm] 8.25 (s, 1.80H), 7.23 (t, *J* = 4.0 Hz, 1H), 6.86 – 6.78 (m, 3H), 3.74 (s, 3H), 2.97 (s, 0.07H); **^13^C NMR** (101 MHz, DMSO-*d*_6_) *δ* [ppm] 159.46, 138.95, 129.70, 120.85, 114.30, 112.24, 55.02; **HR–MS** (ESI-TOF) found 156.1317, theoretical value for C_9_H_9_D_4_NO *m/z* [M+H]^+^ 156.1326.

**^2^H NMR** (400 MHz, DMSO-*d*_6_) *δ* [ppm] 8.04, 3.41, 2.96.

**^1^H NMR** (400 MHz, DMSO-*d*_6_) *δ* [ppm] 8.16 (s, 1.86H), 7.24 – 7.18 (m, 1H), 7.09 – 7.01 (m, 3H), 2.95 (s, 0.11H), 2.28 (s, 3H); **^13^C NMR** (101 MHz, DMSO-*d*_6_) *δ* [ppm] 137.72, 137.24, 129.31, 128.54, 127.37, 125.70, 21.01; **HR–MS** (ESI-TOF) found 140.1372, theoretical value for C_9_H_9_D_4_N *m/z* [M+H]^+^ 140.1377.

**^2^H NMR** (400 MHz, DMSO-*d*_6_) *δ* [ppm] 7.99, 3.38, 2.92.

**^1^H NMR** (400 MHz, DMSO-*d*_6_) *δ* [ppm] 8.13 (s, 2H), 7.37 (q, *J* = 8.0 Hz, 1H), 7.11 (m, 3H), 3.01 (s, 0.09H); **^13^C NMR** (101 MHz, DMSO-*d*_6_) *δ* [ppm] 162.31 (d, *J* = 244.2 Hz), 140.21 (d, *J* = 8.1 Hz), 130.53 (d, *J* = 8.1 Hz), 124.92, 115.56 (d, *J* = 21.2 Hz), 113.59 (d, *J* = 21.2 Hz); **HR–MS** (ESI-TOF) found 144.1121, theoretical value for C_8_H_7_D_4_FN *m/z* [M+H]^+^ 144.1127.

**^2^H NMR** (400 MHz, DMSO-*d*_6_) *δ* [ppm] 7.96, 3.38, 2.99.

**^1^H NMR** (400 MHz, DMSO-*d*_6_) *δ* [ppm] 8.24 (s, 2.06H), 7.53 (t, *J* = 8.0 Hz, 1H), 7.38 (dd, *J*_1_ = 12.0 Hz, *J*_2_ = 4.0 Hz, 1H), 7.20 – 7.11 (m, 1H), 3.00 (s, 0.12H); **^13^C NMR** (101 MHz, DMSO-*d*_6_) *δ* [ppm] 157.16 (d, *J* = 246.4 Hz), 139.24 (d, *J* = 8.1 Hz), 130.63, 126.29, 117.68 (d, *J* = 18.2 Hz), 117.32 (d, *J* = 20.2 Hz); **HR–MS** (ESI-TOF) found 178.0731, theoretical value for C_8_H_5_D_4_ClFN *m/z* [M+H]^+^ 178.0737.

**^2^H NMR** (400 MHz, DMSO-*d*_6_) *δ* [ppm] 8.03, 3.39, 2.96, 2.88.

**^1^H NMR** (400 MHz, DMSO-*d*_6_) *δ* [ppm] 8.40 (s, 3H), 7.48 – 7.42 (m, 2H), 7.21 (td, *J_1_* = 8.0, *J_2_* = 4.0 Hz, 1H), 2.95 (s, 0.19H); **^13^C NMR** (101 MHz, DMSO-*d*_6_) *δ* [ppm] 162.18 (d, *J* = 247.45 Hz), 133.79 (d, *J* = 11.11 Hz), 132.38 (d, *J* = 9.09 Hz), 131.35 (d, *J* = 4.04 Hz), 116.72 (d, *J* = 24.24 Hz), 114.76 (d, *J* = 21.21 Hz); **HR-MS** (ESI-TOF) found 178.0725, theoretical value for C_8_H_7_D_4_FN *m/z* [M+H]^+^ 178.0737.

**^2^H NMR** (400 MHz, DMSO-*d*_6_) *δ* [ppm] 3.99, 2.94.

**^1^H NMR** (400 MHz, DMSO-*d*_6_) *δ* [ppm] 8.22 (d, *J* = 16.0 Hz, 2H), 6.88 (d, *J* = 8.0 Hz, 2H), 6.75 (dd, *J* = 8.0, *J* = 4.0 Hz, 1H), 3.74 (s, 3H), 3.71 (s, 3H), 2.95 (s, 0.08H); **^13^C NMR** (101 MHz, DMSO-*d*_6_) *δ* [ppm] 148.78, 147.62, 129.69, 120.55, 112.46, 111.95, 55.54, 55.45; **HR–MS** (ESI-TOF) found 186.1423, theoretical value for C_10_H_11_D_4_NO_2_ *m/z* [M+H]^+^ 186.1432.

**^2^H NMR** (400 MHz, DMSO-*d*_6_) *δ* [ppm] 8.03, 3.41, 2.92.

**^1^H NMR** (400 MHz, DMSO-*d*_6_) *δ* [ppm] 8.11 (s, 2.95H), 6.43 (d, *J* = 8.0 Hz, 2H), 6.37 (s, 1H), 3.73 (s, 6H), 2.98 (s, 0.15H); **^13^C NMR** (101 MHz, DMSO-*d*_6_) *δ* [ppm] 160.59 (2C), 139.55, 106.68 (2C), 98.53, 55.12 (2C); **HR-MS** (ESI-TOF) found 186.1423, theoretical value for C_10_H_11_D_4_NO_2_ *m/z* [M+H]^+^ 186.1432.

**^2^H NMR** (400 MHz, DMSO-*d*_6_) *δ* [ppm] 3.22, 2.97.

**^1^H NMR** (400 MHz, DMSO-*d*_6_) *δ* [ppm] 8.20 (s, 2.58H), 7.24 (td, *J*_1_ = 8.0 Hz, *J*_2_ = 1.7 Hz, 1H), 7.17 (dd, *J*_1_ = 8.0 Hz, *J*_2_ = 4.0 Hz, 1H), 6.98 (dd, *J*_1_ = 8.0 Hz, *J*_2_ = 4.0 Hz, 1H), 6.89 (t, *J* = 7.4 Hz, 1H), 3.79 (s, 3H), 2.91 (s, 0.17H); **^13^C NMR** (101 MHz, DMSO-*d*_6_) *δ* [ppm] 157.19, 129.97, 128.25, 120.46, 110.83, 55.33; **HR-MS** (ESI-TOF) found 156.1325, theoretical value for C_9_H_9_D_4_NO *m/z* [M+H]^+^ 156.1326.

**^2^H NMR** (400 MHz, DMSO-*d*_6_) *δ* [ppm] 2.82, 3.39.

**^1^H NMR** (400 MHz, DMSO-*d*_6_) *δ* [ppm] 8.22 (s, 2.52H), 7.49 (s, 1H), 7.44 – 7.41 (m, 1H), 7.37 – 7.33 (m, 2H), 3.00 (s, 0.02H); **^13^C NMR** (101 MHz, DMSO-*d*_6_) *δ* [ppm] 131.33, 131.02 (2C), 128.14 (2C), 123.84; **HR-MS** (ESI-TOF) found 141.1320, theoretical value for C_8_H_8_D_4_N_2_ *m/z* [M+H]^+^ 141.1330.

**^2^H NMR** (400 MHz, DMSO-*d*_6_) *δ* [ppm] 8.03, 3.07.

**^1^H NMR** (400 MHz, DMSO-*d*_6_) *δ* [ppm] 8.30 (d, *J* = 12.0 Hz, 1.89H), 7.19 – 7.11 (m, 4H), 2.28 (s, 3H); **^13^C NMR** (101 MHz, DMSO-*d*_6_) *δ* [ppm] 136.09, 135.64, 130.25, 129.09, 126.83, 126.17, 18.90; **HR–MS** (ESI-TOF) found 140.1372, theoretical value for C_9_H_9_D_4_N *m/z* [M+H]^+^ 140.1377.

**^2^H NMR** (400 MHz, DMSO-*d*_6_) *δ* [ppm] 8.11, 3.44, 2.86.

**^1^H NMR** (400 MHz, DMSO-*d*_6_) *δ* [ppm] 8.29 (s, 1.12H), 7.73 – 7.08 (m, 4H), 3.00 (s, 0.09H); **^13^C NMR** (101 MHz, DMSO-*d*_6_) *δ* [ppm] 134.89, 133.11, 131.08, 129.48, 128.91, 127.67; **HR–MS** (ESI-TOF) found 160.0826, theoretical value for C_8_H_6_D_4_ClN *m/z* [M+H]^+^ 160.0831.

**^2^H NMR** (400 MHz, DMSO-*d*_6_) *δ* [ppm] 8.12, 3.41, 2.97.

**^1^H NMR** (400 MHz, DMSO-*d*_6_) *δ* [ppm] 8.19 (s, 2.53H), 6.57 (s, 2H), 3.76 (s, 6H), 3.62 (s, 3H), 3.00 (s, 0.15H); **^13^C NMR** (101 MHz, DMSO-*d*_6_) *δ* [ppm] 152.90 (2C), 136.20, 132.94, 105.99 (2C), 59.91, 55.84 (2C); **HR-MS** (ESI-TOF) found 216.1523, theoretical value for C_11_H_13_D_4_NO_3_ *m/z* [M+H]^+^ 216.1538.

**^2^H NMR** (400 MHz, DMSO-*d*_6_) *δ* [ppm] 2.93, 3.58.

**^1^H NMR** (400 MHz, CD_3_CN-*d*_3_) *δ* [ppm] 7.71 (s, 2.27H), 7.40 – 7.24 (m, 5H), 1.30 (s, 3H), 3.06 (s, 0.04H); **^13^C NMR** (101 MHz, CD_3_CN-*d*_3_) *δ* [ppm] 143.81, 130.33 (2C), 128.81 (2C), 128.65, 20.03 (2C); **HR–MS** (ESI-TOF) found 139.1309, theoretical value for C_9_H_10_D_3_N *m/z* [M+H]^+^ 139.1315.

**^2^H NMR** (400 MHz, CD_3_CN-*d*_3_) *δ* [ppm] 3.20, 3.10.

**^1^H NMR** (400 MHz, DMSO-*d*_6_) *δ* [ppm] 8.23 (d, *J* = 4.0 Hz, 2.04H), 7.51 (t, *J* = 4.0 Hz, 1H), 7.31 (t, *J* = 4.0 Hz, 1H), 7.05 (d, *J* = 4.0 Hz, 1H), 2.99 (s, 0.07H); **^13^C NMR** (101 MHz, DMSO-*d*_6_) *δ* [ppm] 137.48, 128.29, 126.46, 122.08; **HR–MS** (ESI-TOF) found 132.0780, theoretical value for C_6_H_5_D_4_NS *m/z* [M+H]^+^ 132.0785.

**^2^H NMR** (400 MHz, DMSO-*d*_6_) *δ* [ppm] 8.01, 3.39, 2.97, 2.87.

**^1^H NMR** (400 MHz, DMSO-*d*_6_) *δ* [ppm] 8.22 (d, *J* = 20.0 Hz, 2.15H), 6.94 – 6.67 (m, 3H), 5.99 (d, *J* = 12.0 Hz, 2H), 2.93 (s, 0.11H); **^13^C NMR** (101 MHz, DMSO-*d*_6_) *δ* [ppm] 147.42, 146.01, 131.09, 121.77, 109.14, 108.39, 100.88; **HR–MS** (ESI-TOF) found 170.1114, theoretical value for C_9_H_7_D_4_NO_2_ *m/z* [M+H]^+^ 170.1119.

**^2^H NMR** (400 MHz, DMSO-*d*_6_) *δ* [ppm] 8.14, 3.41, 2.96.

**^1^H NMR** (400 MHz, DMSO-*d*_6_) *δ* [ppm] 8.17 (s, 2.33H), 7.25 (m, 5H), 2.73 (s, 0.14H), 2.64 (m, 2H), 1.86 (d, *J* = 8.0 Hz, 1.65H); **^13^C NMR** (101 MHz, DMSO-*d*_6_) *δ* [ppm] 140.98, 128.47 (2C), 128.33 (2C), 126.06, 31.86, 28.56; **HR–MS** (ESI-TOF) found 140.1036, theoretical value for C_8_H_9_D_2_NO *m/z* [M+H]^+^ 140.1044.

**^2^H NMR** (400 MHz, DMSO-*d*_6_) *δ* [ppm] 7.99, 3.43.

**^1^H NMR** (400 MHz, DMSO-*d*_6_) *δ* [ppm] 8.02 (s, 1.43H), 3.35 (t, *J* = 6.5 Hz, 3H), 2.27 (s, 0.08H), 1.77 (t, *J* = 8.0 Hz, 0.31H), 1.48 – 1.43 (m, 2H), 1.35 – 1.27 (m, 2H), 0.87 (t, *J* = 8.0 Hz, 3H); **^13^C NMR** (101 MHz, DMSO-*d*_6_) *δ* [ppm] 69.79, 66.79, 35.02, 31.29, 18.92, 13.85; **HR–MS** (ESI-TOF) found 136.1636, theoretical value for C_7_H_13_D_4_NO *m/z* [M+H]^+^ 136.1639.

**^2^H NMR** (400 MHz, DMSO-*d*_6_) *δ* [ppm] 7.84, 3.51, 1.75.

**^1^H NMR** (400 MHz, DMSO-*d*_6_) *δ* [ppm] 8.62 (s, 1.66H), 7.52 – 7.50 (m, 2H), 7.43 – 7.36 (m, 3H), 3.98 (s, 0.1H); **^13^C NMR** (101 MHz, DMSO-*d*_6_) *δ* [ppm] 133.99, 129.06 (2C), 128.59 (2C), 128.45; **HR–MS** (ESI-TOF) found 110.0934, theoretical value for C_7_H_7_D_2_N *m/z* [M+H]^+^ 110.0939.

**^2^H NMR** (400 MHz, DMSO-*d*_6_) *δ* [ppm] 8.42, 3.97, 3.40.

**^1^H NMR** (400 MHz, DMSO-*d*_6_) *δ* [ppm] 8.50 (t, *J* = 20 Hz, 1.76H), 7.42 (t, *J* = 8.6 Hz, 2H), 6.95 (t, *J* = 8.4 Hz, 2H), 3.90 (s, 0.21H), 3.74 (d, *J* = 8 Hz, 3H); **^13^C NMR** (101 MHz, DMSO-*d*_6_) *δ* [ppm] 159.39, 130.61 (2C), 125.97, 113.94 (2C), 55.23; **HR–MS** (ESI-TOF) found 140.1036, theoretical value for C_8_H_9_D_2_NO *m/z* [M+H]^+^ 140.1044.

**^2^H NMR** (400 MHz, DMSO-*d*_6_) *δ* [ppm] 8.34, 3.88, 3.44.

**^1^H NMR** (400 MHz, DMSO-*d*_6_) *δ* [ppm] 8.54 (s, 2.88H), 7.53 (d, *J* = 8.0 Hz, 2H), 7.40 (t, *J* = 8.0 Hz, 2H), 7.16 (t, *J* = 8.0 Hz, 1H), 7.01 (t, *J* = 8.0 Hz, 4H), 3.96 (s, 0.16H); **^13^C NMR** (101 MHz, DMSO-*d*_6_) *δ* [ppm] 156.79, 156.35, 131.01, 130.09 (2C), 128.94 (2C), 123.67, 118.71 (2C), 118.44 (2C); **HR-MS** (ESI-TOF) found 202.1189, theoretical value for C_13_H_11_D_2_NO *m/z* [M+H]^+^ 202.1201.

**^2^H NMR** (400 MHz, DMSO-*d*_6_) *δ* [ppm] 3.96.

**^1^H NMR** (400 MHz, DMSO-*d*_6_) *δ* [ppm] 8.47 (s, 2.24H), 7.43 (d, *J* = 8.0 Hz, 2H), 7.28 (d, *J* = 8.0 Hz, 2H), 3.93, (s, 0.11H), 2.47 (s, 3H); **^13^C NMR** (101 MHz, DMSO-*d*_6_) *δ* [ppm] 138.75, 130.40, 129.75 (2C), 125.84 (2C), 14.62; **HR–MS** (ESI-TOF) found 156.0810, theoretical value for C_8_H_9_D_2_NS *m/z* [M+H]^+^ 156.0816.

**^2^H NMR** (400 MHz, DMSO-*d*_6_) *δ* [ppm] 8.26, 3.92, 3.42.

**^1^H NMR** (400 MHz, DMSO-*d*_6_) *δ* [ppm] 8.63 (d, *J* = 20.0 Hz, 2.14H), 7.61 (d, *J* = 8.0 Hz, 2H), 7.43 (d, *J* = 8.0 Hz, 2H), 4.00 (s, 0.36H); **^13^C NMR** (101 MHz, DMSO-*d*_6_) *δ* [ppm] 133.79, 132.14, 130.47 (2C), 123.44 (2C); **HR–MS** (ESI-TOF) found 125.1058, theoretical value for C_7_H_8_D_2_N_2_ *m/z* [M+H]^+^ 125.1048.

**^2^H NMR** (400 MHz, DMSO-*d*_6_) *δ* [ppm] 8.44, 4.02.

**^1^H NMR** (400 MHz, DMSO-*d*_6_) *δ* [ppm] 8.48 (s, 2.14H), 7.59 – 7.54 (m, 2H), 7.25 (t, *J* = 8.0 Hz, 2H), 3.98 (s, 0.12H); **^13^C NMR** (101 MHz, DMSO-*d*_6_) *δ* [ppm] 162.14 (d, *J* = 246.4 Hz), 131.44 (d, *J* = 9.1 Hz, 2C), 130.32 (d, *J* = 9.1 Hz), 115.43 (d, *J* = 21.2 Hz, 2C).; **HR–MS** (ESI-TOF) found 128.0838, theoretical value for C_7_H_6_D_2_FN *m/z* [M+H]^+^ 128.0845.

**^2^H NMR** (400 MHz, DMSO-*d*_6_) *δ* [ppm] 8.30, 3.98, 3.42.

**^1^H NMR** (400 MHz, DMSO-*d*_6_) *δ* [ppm] 8.61 (s, 1.97H), 7.57 – 7.51 (m, 2H), 7.47 (d, *J* = 8.0 Hz, 2H), 3.98 (s, 0.19H); **^13^C NMR** (101 MHz, DMSO-*d*_6_) *δ* [ppm] 133.20, 133.08, 131.11 (2C), 128.57 (2C); **HR–MS** (ESI-TOF) found 144.0545, theoretical value for C_7_H_6_D_2_ClN *m/z* [M+H]^+^ 144.0549.

**^2^H NMR** (400 MHz, DMSO-*d*_6_) *δ* [ppm] 8.41, 4.97, 3.44.

**^1^H NMR** (400 MHz, DMSO-*d*_6_) *δ* [ppm] 8.58 (s, 2.16H), 7.31 (t, *J* = 8.0 Hz, 1H), 7.17 (s, 1H), 7.05 (d, *J* = 8.0 Hz, 1H), 6.93 (dd, *J*_1_ = 8.0, *J*_2_ = 4.0 Hz, 1H), 3.95 (s, 0.12H), 3.76 (s, 3H); **^13^C NMR** (101 MHz, DMSO-*d*_6_) *δ* [ppm] 159.35, 135.52, 129.73, 121.02, 114.56, 113.99, 55.23; **HR–MS** (ESI-TOF) found 140.1043, theoretical value for C_8_H_9_D_2_NO *m/z* [M+H]^+^ 140.1044.

**^2^H NMR** (400 MHz, DMSO-*d*_6_) *δ* [ppm] 8.36, 3.93, 3.40.

**^1^H NMR** (400 MHz, DMSO-*d*_6_) *δ* [ppm] 8.65 (s, 2.84H), 7.55 (d, *J* = 8.0 Hz, 1H), 7.52 – 7.45 (m, 2H), 7.38 (d, *J* = 8.0 Hz, 1H), 4.02 (s, 0.23H); **^13^C NMR** (101 MHz, DMSO-*d*_6_) *δ* [ppm] 135.65, 132.75, 129.88, 128.15, 123.37, 123.05; **HR-MS** (ESI-TOF) found 125.1041, theoretical value for C_7_H_8_D_2_N_2_ *m/z* [M+H]^+^ 125.1048.

**^2^H NMR** (400 MHz, DMSO-*d*_6_) *δ* [ppm] 4.00.

**^1^H NMR** (400 MHz, DMSO-*d*_6_) *δ* [ppm] 8.43 (s, 3H), 7.31 – 7.28 (m, 3H), 7.24 – 7.15 (m, 1H), 3.95 (s, 0.2H), 2.31 (s, 3H); **^13^C NMR** (101 MHz, DMSO-*d*_6_) *δ* [ppm] 137.68, 133.86, 129.47, 128.96, 128.47, 125.94, 20.91; **HR-MS** (ESI-TOF) found 124.1088, theoretical value for C_8_H_9_D_2_N *m/z* [M+H]^+^ 124.1095.

**^2^H NMR** (400 MHz, DMSO-*d*_6_) *δ* [ppm] 3.94.

**^1^H NMR** (400 MHz, DMSO-*d*_6_) *δ* [ppm] 8.48 (s, 2.66H), 7.49-7.44 (m, 1H), 7.40 (dt, *J*_1_ = 12.0, *J*_2_ = 4.0 Hz, 1H), 7.34 (d, *J* = 8.0 Hz, 1H), 7.22 (td, *J*_1_ = 8.0, *J*_2_ = 2.0 Hz, 1H), 4.02 (s, 0.2H); **^13^C NMR** (101 MHz, DMSO-*d*_6_) *δ* [ppm] 163.17 (d, *J* = 244.4 Hz), 136.74, 130.60 (d, *J* = 8.08 Hz), 125.11 (d, *J* = 3.03 Hz), 115.94 (d, *J* = 22.22 Hz), 115.29 (d, *J* = 21.21 Hz); **HR-MS** (ESI-TOF) found 128.0837, theoretical value for C_7_H_6_D_2_FN *m/z* [M+H]^+^ 128.0845.

**^2^H NMR** (400 MHz, DMSO-*d*_6_) *δ* [ppm] 4.03.

**^1^H NMR** (400 MHz, DMSO-*d*_6_) *δ* [ppm] 8.50 (s, 3H), 7.26 (d, *J* = 1.9 Hz, 1H), 7.00 (dd, *J_1_* = 8.0, *J_2_* = 1.9 Hz, 1H), 6.94 (d, *J* = 8.0 Hz, 1H), 3.94 (s, 0.02H), 3.76 (s, 3H), 3.74 (s, 3H); **^13^C NMR** (101 MHz, DMSO-*d*_6_) *δ* [ppm] 148.85, 148.61, 126.22, 121.44, 113.00, 111.58, 55.57 (2C); **HR-MS** (ESI-TOF) found 170.1155, theoretical value for C_9_H_11_D_2_NO_2_ *m/z* [M+H]^+^ 170.1150.

**^2^H NMR** (400 MHz, DMSO-*d*_6_) *δ* [ppm] 4.88.

**^1^H NMR** (400 MHz, DMSO-*d*_6_) *δ* [ppm] 8.50 (s, 2.09H), 7.42 (d, *J* = 8.0 Hz, 1H), 7.31 – 7.19 (m, 3H), 3.97 (s, 0.11H), 2.35 (s, 3H); **^13^C NMR** (101 MHz, DMSO-*d*_6_) *δ* [ppm] 136.76, 130.37, 129.26, 128.57, 126.11 (2C), 18.85; **HR–MS** (ESI-TOF) found 124.1091, theoretical value for C_8_H_9_D_2_N *m/z* [M+H]^+^ 124.1095.

**^2^H NMR** (400 MHz, DMSO-*d*_6_) *δ* [ppm] 8.25, 3.96, 3.41.

**^1^H NMR** (400 MHz, DMSO-*d*_6_) *δ* [ppm] 8.50 (d, *J* = 16.0 Hz, 2.28H), 7.14 (d, *J* = 4.0 Hz, 1H), 7.02 – 6.90 (m, 2H), 3.89 (s, 0.19H), 6.03 (s, 2H); **^13^C NMR** (101 MHz, DMSO-*d*_6_) *δ* [ppm] 147.30 (2C), 127.63, 122.97, 109.57, 108.30, 101.24; **HR–MS** (ESI-TOF) found 154.0834, theoretical value for C_8_H_7_D_2_NO_2_ *m/z* [M+H]^+^ 154.0837.

**^2^H NMR** (400 MHz, DMSO-*d*_6_) *δ* [ppm] 8.27, 3.88, 3.42.

**^1^H NMR** (400 MHz, DMSO-*d*_6_) *δ* [ppm] 8.58 (s, 2.04H), 8.03 – 7.87 (m, 4H), 7.65 (dd, *J*_1_ = 8.0, *J*_1_ = 1.5 Hz, 1H), 7.59 – 7.52 (m, 2H), 4.16 (s, 0.18H); **^13^C NMR** (101 MHz, DMSO-*d*_6_) *δ* [ppm] 132.68, 132.64, 128.27, 128.06, 127.82 (2C), 127.74 (2C), 126.68, 126.64; **HR–MS** (ESI-TOF) found 160.1090, theoretical value for C_11_H_9_D_2_N *m/z* [M+H]^+^ 160.1095.

**^2^H NMR** (400 MHz, DMSO-*d*_6_) *δ* [ppm] 8.41, 4.14, 3.42.

**^1^H NMR** (400 MHz, DMSO-*d*_6_) *δ* [ppm] 10.87 (d, *J* = 16.0 Hz, 0.79H), 8.21 (s, 2.6H), 7.30 – 7.21 (m, 1H), 7.21 – 7.13 (m, 1H), 7.08 (d, *J* = 16.0 Hz, 1H), 6.78 – 6.56 (m, 1H), 3.74 (d, *J* = 16.0 Hz, 3H), 2.98 (s, 0.18H); **^13^C NMR** (101 MHz, DMSO-*d*_6_) *δ* [ppm] 153.16, 131.47, 127.24, 123.89, 112.29, 111.33, 109.25, 100.18, 55.49; **HR–MS** (ESI-TOF) found 195.1426, theoretical value for C_11_H_10_D_4_N_2_O *m/z* [M+H]^+^ 195.1435.

**^2^H NMR** (400 MHz, DMSO-*d*_6_) *δ* [ppm] 8.05, 3.45.

**^1^H NMR** (400 MHz, CDCl_3_) *δ* [ppm] 8.39 (s, 1.23H), 7.24 (s, 1H), 7.02 (s, 1H), 6.98 (s, 1H), 6.85 (d, *J* = 12.0 Hz, 1H), 3.84 (s, 3H), 2.91 (s, 0.18H), 1.92 (s, 3H); **^13^C NMR** (101 MHz, CDCl_3_) *δ* [ppm] 170.61, 153.87, 131.49, 127.60, 120.98, 112.25 (2C), 112.03, 100.27, 55.84, 23.21; **HR–MS** (ESI-TOF) found 237.1531, theoretical value for C_13_H_12_D_4_N_2_O_2_ *m/z* [M+H]^+^ 237.1541.

**^2^H NMR** (400 MHz, CDCl_3_) *δ* [ppm] 3.55, 2.89.

**^1^H NMR** (400 MHz, DMSO-*d*_6_) *δ* [ppm] 11.05 (s, 0.46H), 8.23 (s, 1.49H), 7.57 (d, *J* = 8.0 Hz, 1H), 7.37 (d, *J* = 12.0 Hz, 1H), 7.24 (s, 1H), 7.07 (t, *J* = 8.0 Hz, 1H), 6.99 (d, *J* = 8.0 Hz, 1H), 3.00 (s, 0.19H); **^13^C NMR** (101 MHz, DMSO-*d*_6_) *δ* [ppm] 136.33, 126.87, 123.38, 121.18, 118.48, 118.17, 111.58, 109.44; **HR–MS** (ESI-TOF) found 165.1320, theoretical value for C_10_H_8_D_4_N_2_ *m/z* [M+H]^+^ 165.1330.

**^2^H NMR** (400 MHz, DMSO-*d*_6_) *δ* [ppm] 7.99, 3.63.

**^1^H NMR** (400 MHz, DMSO-*d*_6_) *δ* [ppm] 10.75 (s, 0.56H), 7.34 (d, *J* = 8.0 Hz, 2H), 7.26 (d, *J* = 8.0 Hz, 2H), 6.96 (dt, *J*_1_ = 32, *J*_2_ = 8.0 Hz, 2H), 2.98 – 1.67 (m, 10H); **^13^C NMR** (101 MHz, DMSO-*d*_6_) *δ* [ppm] 141.02, 135.53, 126.80, 120.53, 118.24, 117.63, 110.95, 105.93, 52.74, 35.19 (2C), 25.28, 20.83 (2C).

**^2^H NMR** (400 MHz, DMSO-*d*_6_) *δ* [ppm] 3.50.

**^1^H NMR** (400 MHz, DMSO-*d*_6_) *δ* [ppm] 7.39 (s, 0.57H), 7.16 (d, *J* = 8.0 Hz, 2H), 6.85 (d, *J* = 8.0 Hz, 2H), 3.72 (s, 3H), 3.27 (s, 2H); **^13^C NMR** (101 MHz, DMSO-*d*_6_) *δ* [ppm] 172.63, 157.86, 130.05 (2C), 128.46, 113.60 (2C), 55.04, 41.37.

**^1^H NMR** (400 MHz, DMSO-*d*_6_) *δ* [ppm] 7.17 (t, *J* = 8.0 Hz, 2H), 6.87 (t, *J* = 8.0 Hz, 2H), 3.73 (d, *J* = 8.0 Hz, 3H), 3.48 (d, *J* = 8.0 Hz, 2H); **^13^C NMR** (101 MHz, DMSO-*d*_6_) *δ* [ppm] 173.03, 158.04, 130.41 (2C), 126.97, 113.68 (2C), 55.04, 40.15.

**Supplementary References**

1. Wang, Z., Guo, X., Montoya, J. & Nørskov, J. K. Predicting aqueous stability of solid with computed Pourbaix diagram using SCAN functional. *npj Comput. Mater.* **6**, 160 (2020).
2. Flak, D. et al. In situ ambient pressure XPS observation of surface chemistry and electronic structure of *α*-Fe_2_O_3_ and *γ*-Fe_2_O_3_ nanoparticles. *Appl. Surf. Sci.* **455**, 1019−1028 (2018).
3. Iqbal, M. et al. Continuous mesoporous Pd films by electrochemical deposition in nonionic micellar solution. *Chem. Mater.* **29**, 6405−6413 (2017).
4. Kresse, G & Furthmüller, J. Efficiency of Ab-initio total energy calculations for metals and semiconductors using a plane-wave basis set. *Comput. Mater. Sci.* **6**, 15−50 (1996).
5. Kresse, G. & Furthmüller, J. Efficient iterative schemes for Ab initio total-energy calculations using a plane-wave basis set. *Phys. Rev. B* **54**, 11169−11186 (1996).
6. Perdew, J. P., Burke, K. & Ernzerhof, M. Generalized gradient approximation made simple. *Phys. Rev. Lett.* **77**, 3865−3868 (1996).
7. Dudarev, S. L., Botton, G. A., Savrasov, S. Y., Humphreys, C. J. & Sutton, A. P. Electron-energy-loss spectra and the structural stability of nickel oxide: an LSDA+U study. *Phys. Rev. B* **57**, 1505−1509 (1998).
8. Xu, H., Cheng, D., Cao, D. & Zeng, X. A universal principle for a rational design of single-atom electrocatalysts. *Nat. Catal.* **1**, 339−348 (2018).
9. Grimme, S., Antony, J., Ehrlich, S. & Krieg, S. A consistent and accurate ab initio parametrization of density functional dispersion correction (DFT-D) for the 94 elements H-Pu. *J. Chem. Phys.* **132**, 154104 (2010).
10. Nørskov, J., Rossmeisl, J., Logadottir, A. & Lindqvist, L. Origin of the overpotential for oxygen reduction at a fuel-cell cathode. *J. Phys. Chem. B* **108**, 17886−17892 (2004).
11. Wang, X. et al. General and practical potassium methoxide/disilane-mediated dehalogenative deuteration of (hetero)arylhalides. *J. Am. Chem. Soc.* **140**, 10970−10974 (2018).
12. Pony Yu, R., Hesk, D., Rivera, N., Pelczer, I. & Chirik, P. J. Iron-catalysed tritiation of pharmaceuticals. *Nature* **529**, 195−199 (2016).
13. Loh, Y. Y. et al. Photoredox-catalyzed deuteration and tritiation of pharmaceutical compounds. *Science* **358**, 1182−1187 (2017).
14. Xie, J., Yan, P., Zhang, Q., Yuan, K. & Zhou Q. Asymmetric hydrogenation of cyclic imines catalyzed by chiral spiro iridium phosphoramidite complexes for enantioselective synthesis of tetrahydroisoquinolines. *ACS Catal.* **2**, 561−564 (2012).
15. Zeng, H., Wang, Z. & Li, C.-J. Two-in-one strategy for palladium-catalyzed C−H Functionalization in Water. *Angew. Chem. Int. Ed.* **58**, 2859–2863 (2019).
